# A prospective cohort study on the association of lean body mass estimated by mid‐upper arm muscle circumference with hypertension risk in Chinese residents

**DOI:** 10.1111/jch.14412

**Published:** 2022-02-16

**Authors:** Yuyan Liu, Guifan Sun, Yongfang Li

**Affiliations:** ^1^ Department of Clinical Epidemiology The Fourth Affiliated Hospital of China Medical University Shenyang Liaoning China; ^2^ Research Center of Environmental and Non‐Communicable Disease School of Public Health China Medical University Shenyang Liaoning China

**Keywords:** blood pressure, hypertension, lean body mass, mid‐upper arm muscle circumference

## Abstract

The associations of lean body mass (LBM) with elevated blood pressure (BP) and hypertension were controversial, and the causalities have never been shown. Mid‐upper arm muscle circumference (MAMC), an easily obtained anthropometric measurement, could provide an accurate estimate for LBM. Therefore, a prospective cohort study in general Chinese residents aiming to find out the relationship between LBM estimated using MAMC and hypertension risk was performed. Eight thousand one hundred eighty‐five eligible participants were included in the baseline analysis, among whom 3442 were subsequently selected into cohort analysis. MAMC was calculated using mid‐upper arm circumference (MUAC) and triceps skinfold thickness (TST). Associations of MAMC with BP values and hypertension prevalence were estimated by linear and logistic regression models. Associations with hypertension incidence were estimated by COX regression models, hazard ratio (HR) and 95% confidence interval (CI) were given. Nonlinear relationship between MAMC and hypertension risk was estimated using restricted cubic spline method. Standardized coefficients of MUAC and TST were compared to estimate their strengths of associations with hypertension. Baseline analysis showed that after adjusted for confounders, the increase of systolic BP per standard deviation (SD) of MAMC were 1.97 mmHg (95%CI: 1.46, 2.48) and 1.63 mmHg (95%CI: 1.10, 2.16) respectively in men and women, and the increases of diastolic BP per SD were 1.58 mmHg (95%CI: 1.23, 1.92) and 1.08 mmHg (95%CI: 0.74, 1.42). Additionally, the association of MAMC with the prevalence of hypertension were also found in both men and women (OR = 1.36, 95%CI: 1.26, 1.47 in men; OR = 1.33, 95%CI: 1.22, 1.44 in women). Cohort analysis showed that MAMC increased the risk of hypertension (HR = 1.10, 95%CI: 1.01, 1.19 for men; HR = 1.15, 95%CI: 1.06, 1.26 for women), and a trend of J‐shaped relationship was found. Additionally, the stronger associations of MUAC with both BP values and hypertension than that of TST were found in both baseline and cohort analyses. Findings in our study implied that we cannot neglect the capacity of LBM in predicting hypertension risk, and LBM estimates should be recommended in general health surveys or examinations.

## 1 INTRODUCTION

Lean body mass (LBM) or skeletal muscle mass plays a key role in energy metabolism, and the promotion of LBM and muscular strength is always advocated in preventing obesity‐related metabolic abnormalities, as well as geriatric sarcopenia and frailty.^1^, ^2^, ^3^ Elevated blood pressure (BP) and hypertension are major risk factors of several cardiovascular diseases (CVDs), and generate huge health and economic burdens.^4^, ^5^, ^6^ The unfavorable relationship between LBM and hypertension has been found in cross‐sectional studies, while opposite findings were also reported.^7^, ^8^, ^9^, ^10^ Such inconsistency was probably resulted from the variances of populations, as well as the utilities of different measurements of LBM. On the other hand, the deficiency of longitudinal design in published studies made it failed to verify the causality between increased LBM and hypertension.

Currently, accurate measurements of LBM are majorly based on image methods, such as dual‐energy x‐ray absorptiometry (DXA) and computed tomography (CT). However, high costs and complicated operations limit their wide utilities among general populations.^11^ Mid‐upper arm muscle circumference (MAMC) is a novel anthropometric index of LBM (skeletal muscle majorly) calculated using mid‐upper arm circumference (MUAC) and triceps skinfold thickness (TST), both of which respectively reflected the overall amount of muscle and fat, and the subcutaneous fat of mid‐upper arm.^12^, ^13^, ^14^ As an easier obtained measurement, MAMC has been proven to be closely correlated to some other more accurate measurements based on DXA and CT in various populations.^15^, ^16^, ^17^ However, studies about the relationship between MAMC and cardiovascular abnormalities are limited.^18^, ^19^

In addition, despite that positive associations of both MUAC and TST with hypertension have been reported, no study has disclosed which one was stronger associated with hypertension.^20^, ^21^ If the positive relationship between LBM and hypertension existed, MUAC, as a measurement of both fat and lean mass perhaps should be stronger associated with hypertension than TST. Such comparative analysis we thought would be necessary to indirectly verify the relationship between LBM and hypertension.

Therefore, in this study, we aimed to find out if MAMC, as an easier obtained anthropometric surrogate of LBM was positively associated with hypertension risk based on a large‐scale longitudinal study among Chinese population; and also compare strengths of associations with hypertension between MUAC and TST to further elucidate the relationship between LBM and hypertension.

## 2 METHODS

### 2.1 Study design and populations

**FIGURE 1:**
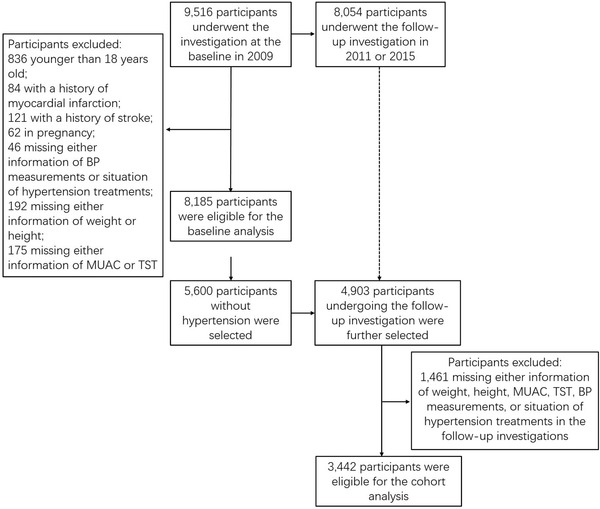
Flow chart of this study

The China Health and Nutrition Survey (CHNS) is an ongoing open cohort project aiming to examine the effects of social and economic transformation of Chinese society on nutrition and health behaviors and outcomes among general Chinese residents.^22^ CHNS started from 1989 including 15 907 participants, and 10 rounds of follow‐up surveys in 15 provinces and municipal cities have been completed by 2015.^23^ Considering that the biospecimens were collected from 2009, three waves of surveys (ie, 2009, 2011, 2015) were only included in our analysis. As shown in Figure 1, there were 9516 participants undergoing the baseline investigation in 2009, among whom 8054 were investigated in the follow‐up surveys of 2011 or 2015 (39 736 person‐years in total). We firstly excluded participants who were not eligible for the following analyses, including 836 younger than 18 years old; 84 with a history of myocardial infarction; 121 with a history of stroke; 62 in pregnancy; 46 missing either information of BP measurements or situation of hypertension treatments; 192 missing either information of weight or height; and 175 missing either information of MUAC or TST. As a result, 8185 participants were finally remained for the baseline analysis, among whom 5600 were without hypertension. By further excluding those without relevant follow‐up information, 3442 participants were eligible for the cohort analysis. The data of CHNS are publicly available, and the researchers from the National Institute for Nutrition and Health, Chinese Center for Disease Control and Prevention and the Carolina Population Center, University of North Carolina at Chapel Hill had received ethic approval in the Institute Review Board.

### 2.2 Measurements of MUAC and TST

The mid‐point of the mid‐upper arm was defined as the midway between the olecranon process of the ulna and the acromion process of the scapula, which was located after bending the right arm to a 90° angle at the elbow. MUAC was measured at the mid‐point of mid‐upper arm with the elbow fully extended using a metric scale, and recorded results to the nearest 0.1 cm. TST was measured at the halfway between the acromion process and the olecranon process, while the arm was hanging relaxed at the participant's side. The fold of skin and underlying subcutaneous fat 2.0 cm above the place where the measurement was to be taken were firstly grasped using the thumb and index finger, and then measured using a skinfold caliper with a constant pressure of 10 g/mm.^2^ The jaws of calipers were placed at the marked level, perpendicular to the skinfold, and the measurement was read within 3 s and to the nearest 0.5 mm. All measurements were performed by trained health care workers following the World Health Organization‐recommended protocols.^20^, ^24^ For both MUAC and TST, 3‐time measurements were respectively obtained per participant on the right arm as the first choice, and the average were used for the following analyses.

MAMC was calculated using the standard formula: MAMC (cm) = MUAC (cm) – π * TST (mm) /10.^24^, ^25^

### 2.3 BP measurements and the definition of hypertension

BP was measured using a standard mercury‐column sphygmomanometer with an appropriate adult upper arm cuff size after 5 min of rest in the sitting position based on the standardized procedural guideline. Systolic blood pressure (SBP) and diastolic blood pressure (DBP) were determined by the first and the fifth Korotkoff sounds. BP on both right and left arm was firstly measured, and the arm with higher blood pressure would be selected to obtain three consecutive measurements with a time interval of at least 1 min. Then, the average on the selected arm would be recorded as the final BP. Before measuring, participants were also need to be asked for not doing following behaviors: drinking alcohol, tea or coffee; smoking; or taking any exercise for at least 30 min before measuring BP.^26^, ^27^ In this study, we defined hypertension as SBP/DBP ≥ 140/90 mmHg or antihypertensive medication use.

### 2.4 Measurements of confounders

All participants were asked to undergo body weight and height measurements while wearing light clothes without shoes. Body weight and height were measured using a vertical weight scale and the metric scale with a standardized protocol, respectively. Both body weight and height were measured to the nearest 0.1 kg and 0.1 cm, respectively. BMI was then calculated as weight (kg) divided by the square of the height (m).

Blood samples (12 mL) were collected after at least 8 h of overnight fasting, and then transferred to the local hospital for further treatment within 2 h of collection. The blood samples in red‐stoppered tube (4 mL) were centrifuged at 3000 × g for 15 min at room temperature; serum samples were frozen and stored at −86 °C for the subsequent laboratory analysis. According to strict quality control standards, all samples were verified and analyzed in a national central laboratory in Beijing (Medical laboratory accreditation certificate: ISO 15189:2007). Triglycerides (TG) was measured using glycerol‐phosphate oxidase (GPO‐PAP) method (Kyowa, Tokyo, Japan). High density lipid‐cholesterol (HDL‐C) was measured using enzymatic methods (Kyowa), and total cholesterol (TC) was measured by the cholesterol oxidase‐phenol and aminophenazone (CHOD‐PAP) method. Fasting glucose (FG) was measured by glucose oxidase‐phenol and aminophenazone (GOD‐PAP) method (Randox, Crumlin, UK). All biochemical assessment aforementioned were performed using Hitachi 7600 automated analyzer (Hitachi Inc., Tokyo, Japan).^28^, ^29^

In addition, self‐administered questionnaires were also used to obtain information on demographic characteristics, medical history, medication use, smoking habits, and other pertinent factors. Trained staff members confirmed the reported information with each participant. In this study, smoking status was categorized as current smokers, past smokers, and never. Drinking status were categorized as current drinkers and never.

### 2.5 Statistical analysis

All continuous variables were presented as mean and standard deviation (SD). Categorical variables were presented as percentages. Student t test and chi‐square test were respectively used to estimate the difference of continuous and categorical variables between men and women.

In the baseline analysis, multivariable linear regression analyses were used to estimate the association of BP (SBP and DBP) with per SD increase of MAMC, MUAC and TST in participants without any antihypertensive medication use. The associations of hypertension with MAMC, MUAC and TST were estimated using multivariable logistic regression models, and results were shown using odds ratio (OR) and 95% confidence interval (95% CI).

In the cohort analysis, associations of changes of BP values during the follow‐up with MAMC, MUAC, and TST were firstly estimated using linear regression models. We then used COX regression models to estimate the hypertension risk attributed to MAMC, MUAC and TST, and results were shown as hazard ratio (HR) and 95% CI. Restricted cubic splines (RCS) analyses with three knots at the 10th, 50th, and 90th centiles of MAMC, MUAC and TST were also used to estimate their associations with hypertension with flexibility by running rms package in R (v.4.0.3) software, and the values of their medians were set as the reference. On the other hand, associations of changes of MAMC, MUAC and TST during the follow‐up with both changes of BP values and hypertension risk were estimated. We performed all above analyses in 3 models: Model 1 (adjusted for age), Model 2 (adjusted for smoking and alcohol drinking in addition to Model 1), and Model 3 (adjusted for TG, TC, HDL‐C, and FG in addition to Model 1). All analyses were respectively completed in both men and women, and P‐values of interaction by gender were given.

All above statistical analyses were completed using SAS 9.4 (SAS Institute, Inc., Cary, NC, USA) expect those with special notes. *p*‐Value < .05 was considered as statistical significance.

## 3 RESULTS

### 3.1 General characteristics

Characteristics of the 8,185 participants (3,819 men) are shown in Table 1 as mean ± SD and percentages. No difference of age or BMI was found between men and women. Compared to women, larger MAMC and MUAC, as well as smaller TST were found in men with statistical significance. Both BP values and prevalence of hypertension were higher in men. Among biochemical biomarkers, higher FG and TG, but lower TC and HDL‐C were found in men. In addition, men also showed dramatically higher percentages of cigarette smoking and alcohol drinking than women.

**TABLE 1:** Baseline characteristics of participants (no. = 8185)

	Men (n=3,819)	Women (n=4,366)	P‐value
Age (years old)	50.2 ± 15.1	50.5 ± 14.8	0.275
Weight (kg)	65.3 ± 11.4	56.9 ± 9.7	<0.001
Height (cm)	167.0 ± 6.7	160.0 ± 6.5	<0.001
BMI (kg/m^2^)	23.3 ± 3.4	23.4 ± 3.5	0.616
MAMC (cm)	23.1 ± 3.4	21.0 ± 3.2	<0.001
MUAC (cm)	27.7 ± 3.6	26.6 ± 3.6	<0.001
TST (mm)	14.6 ± 7.7	18.1 ± 7.2	<0.001
SBP (mmHg)	125.8 ± 17.3	123.4 ± 19.9	<0.001
DBP (mmHg)	82.1 ± 11.1	79.1 ± 11.3	<0.001
FG (mmol/L)	5.5 ± 1.6	5.3 ± 1.3	<0.001
TG (mmol/L)	1.8 ± 1.7	1.5 ± 1.2	<0.001
TC (mmol/L)	4.8 ± 1.0	4.9 ± 1.0	<0.001
HDL‐C (mmol/L)	1.4 ± 0.5	1.5 ± 0.5	<0.001
Hypertension (%)	32.1	27.9	<0.001
Antihypertensive medication (%)	8.6	10.3	0.012
Smoking (%)			<0.001
Current	55.6	3.7	
Past	6	0.4	
Never	38.4	96	
Alcohol drinking (%)	60.7	8.9	<0.001

Values are presented as mean ± SD, or %. Abbreviations: BMI, body mass index; MAMC, mid‐upper arm muscle circumference; MUAC, mid‐upper arm circumference; TST, triceps skinfold thickness; SBP, systolic blood pressure; DBP, diastolic blood pressure; FG, fasting glucose; TG, triglycerides; TC, total cholesterol; HDL‐C, high‐density lipid cholesterol.

### 3.2 Associations of MAMC, MUAC and TST with BP and hypertension prevalence at baseline

Among 7,406 participants without any antihypertensive treatment at baseline, SBP was independently associated with MAMC in both men and women, and the increase of SBP per SD of MAMC were 1.97  and 1.63 mmHg respectively in men and women after adjusted for age and biochemical markers in Model 3 (Table 2). The independently positive association of SBP with MUAC was found in both men and women, while for TST, the statistically significant association was only found in women. Statistically significant *p*‐value for interaction by gender was found for TST (*p*‐value = .014). Consistent results were found for DBP. As shown in Table 2, the increases per SD of MAMC were 1.58  and 1.08 mmHg respectively in men and women in Model 3. The positive association of DBP with MUAC was found in both men and women, while the association of DBP with TST was only found in women in Model 3, and *p*‐value for interaction by gender was .012. Compared with TST, MUAC showed stronger association with BP due to a larger value of BP increase per SD of MUAC in both men and women.

**TABLE 2:** Adjusted associations of blood pressure with per SD increase of MAMC, MUAC, and TST (no. = 7406)

	Men (n=3,489)			Women (n=3,917)			
	BP (mmHg)	95% CI	P‐value	BP (mmHg)	95% CI	P‐value	P‐value for interaction
SBP							
Model 1							
MAMC	2.15	1.65, 2.66	<0.001	1.93	1.40, 2.46	<0.001	0.440
MUAC	2.65	2.14, 3.17	<0.001	2.66	2.17, 3.15	<0.001	0.536
TST	0.76	0.26, 1.26	0.003	1.79	1.27, 2.31	<0.001	0.044
Model 2							
MAMC	2.12	1.61, 2.63	<0.001	1.93	1.40, 2.46	<0.001	0.458
MUAC	2.61	2.09, 3.12	<0.001	2.66	2.17, 3.15	<0.001	0.537
TST	0.7	0.20, 1.21	0.006	1.8	1.28, 2.32	<0.001	0.040
Model 3							
MAMC	1.97	1.46, 2.48	<0.001	1.63	1.10, 2.16	<0.001	0.622
MUAC	2.37	1.84, 2.89	<0.001	2.28	1.78, 2.78	<0.001	0.978
TST	0.46	‐0.04, 0.97	0.072	1.45	0.93, 1.98	<0.001	0.014
DBP							
Model 1							
MAMC	1.74	1.39, 2.08	<0.001	1.33	0.99, 1.67	<0.001	0.074
MUAC	2.52	2.17, 2.87	<0.001	2.15	1.84, 2.46	<0.001	0.047
TST	1.15	0.80, 1.49	<0.001	1.78	1.45, 2.11	<0.001	0.047
Model 2							
MAMC	1.68	1.33, 2.03	<0.001	1.33	0.99, 1.67	<0.001	0.098
MUAC	2.44	2.09, 2.79	<0.001	2.15	1.84, 2.47	<0.001	0.067
TST	1.08	0.73, 1.42	<0.001	1.78	1.45, 2.11	<0.001	0.034
Model 3							
MAMC	1.58	1.23, 1.92	<0.001	1.08	0.74, 1.42	<0.001	0.124
MUAC	2.28	1.93, 2.64	<0.001	1.84	1.53, 2.16	<0.001	0.190
TST	0.90	0.55, 1.24	<0.001	1.51	1.18, 1.85	<0.001	0.012

MAMC, MUAC, and TST were analyzed in separate regression models. Model 1: adjusted for age; Model 2: adjusted for smoking and alcohol drinking in addition to Model 1; Model 3: adjusted for TG, TC, HDL‐C, and FG in addition to Model 1. Abbreviations: BP, blood pressure; 95% CI, 95% confidence interval; SBP, systolic blood pressure; DBP, diastolic blood pressure; MAMC, mid‐upper arm muscle circumference; MUAC, mid‐upper arm circumference; TST, triceps skinfold thickness.

As shown in Table 3, the association of MAMC with the prevalence of hypertension were found in both men and women in Model 3 (OR = 1.36, 95%CI: 1.26, 1.47 in men; OR = 1.33, 95%CI: 1.22, 1.44 in women), and no interaction by gender was found (Table 3). Also, positive associations of MUAC and TST with hypertension were found, respectively. Compared with TST, MUAC showed stronger association with the prevalence of hypertension with a larger OR per SD of MUAC in both men and women.

**TABLE 3:** Adjusted associations of hypertension prevalence with per SD increase of MAMC, MUAC, and TST (no. = 8185)

	Men (n=3,819)			Women (n=4,366)			
	OR	95% CI	P‐value	OR	95% CI	P‐value	P‐value for interaction
Model 1							
MAMC	1.41	1.30, 1.52	<0.001	1.39	1.28, 1.50	<0.001	0.193
MUAC	1.59	1.47, 1.72	<0.001	1.62	1.49, 1.75	<0.001	0.555
TST	1.19	1.11, 1.28	<0.001	1.39	1.28, 1.50	<0.001	0.111
Model 2							
MAMC	1.40	1.29, 1.51	<0.001	1.39	1.28, 1.51	<0.001	0.210
MUAC	1.58	1.46, 1.71	<0.001	1.62	1.50, 1.75	<0.001	0.538
TST	1.18	1.10, 1.27	<0.001	1.39	1.28, 1.50	<0.001	0.098
Model 3							
MAMC	1.36	1.26, 1.47	<0.001	1.33	1.22, 1.44	<0.001	0.381
MUAC	1.52	1.41, 1.65	<0.001	1.53	1.41, 1.66	<0.001	0.305
TST	1.15	1.07, 1.24	<0.001	1.33	1.23, 1.44	<0.001	0.048

MAMC, MUAC, and TST were analyzed in separate regression models. Model 1: adjusted for age; Model 2: adjusted for smoking and alcohol drinking in addition to Model 1; Model 3: adjusted for TG, TC, HDL‐C, and FG in addition to Model 1. Abbreviations: OR, odds ratio; 95% CI, 95% confidence interval; MAMC, mid‐upper arm muscle circumference; MUAC, mid‐upper arm circumference; TST, triceps skinfold thickness.

### 3.3 Associations of MAMC, MUAC, and TST with changes of BP value during the follow‐up and hypertension risk in cohort analysis

**FIGURE 2:**
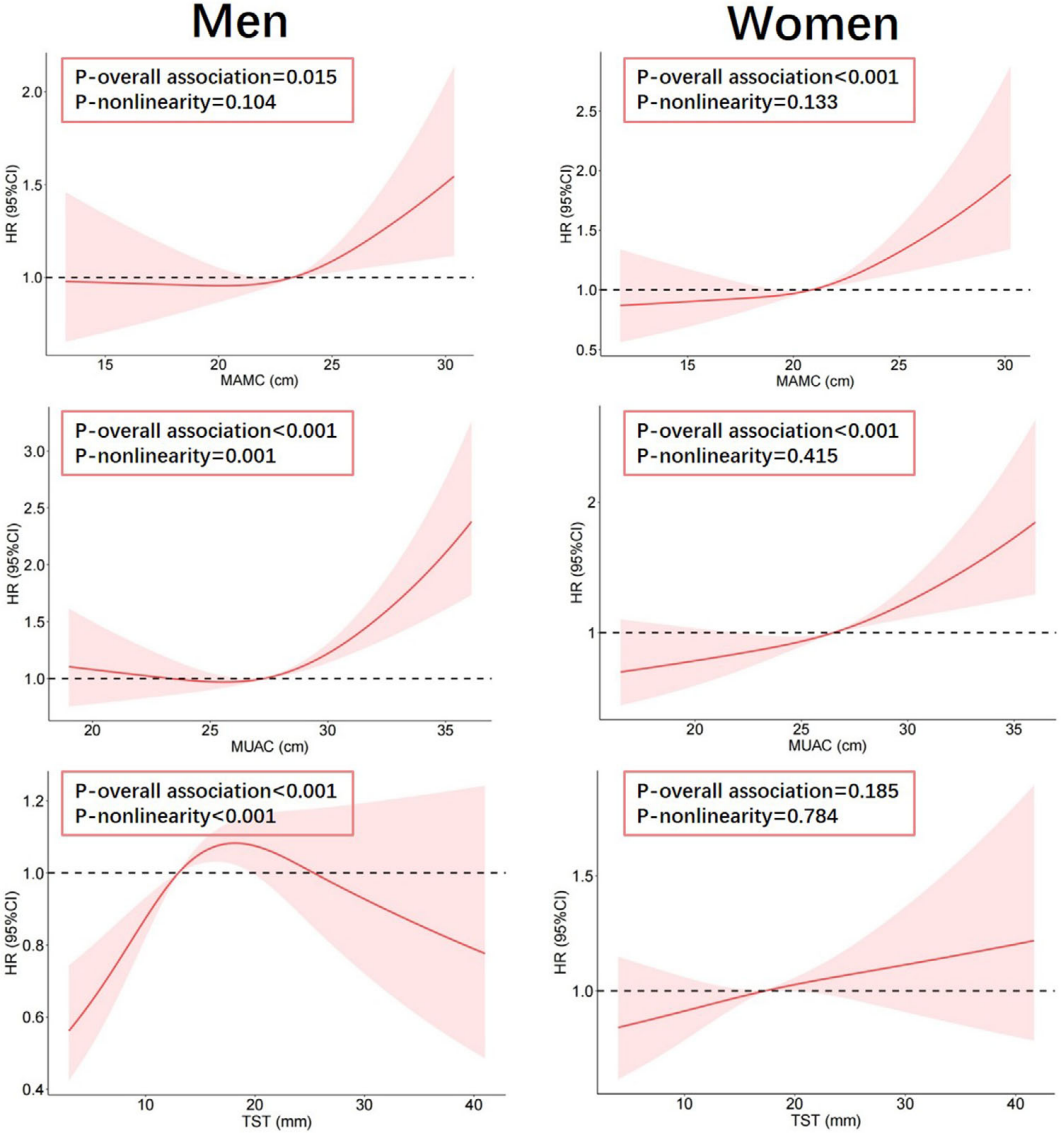
RCS results reflecting nonlinear associations of hypertension risk with MAMC, MUAC, and TST. Three knots at the 10th, 50th, and 90th centiles of MAMC, MUAC, and TST were used, and the values of medians were set as references. All HRs in A, B, and C were adjusted for age, TG, TC, HDL‐C, and FG. Abbreviations: RCS, restricted cubic spline; HR, hazard ratio; 95% CI, 95% confidence interval; MAMC, mid‐upper arm muscle circumference; MUAC, mid‐upper arm circumference; TST, triceps skinfold thickness; BMI, body mass index

Results of cohort analysis showed that the change of SBP during the follow‐up were only associated with MUAC and TST in men in Model 2 (*p*‐values were .049 and .029 for MUAC and TST, respectively), while such associations were abolished in Model 3 (Table S1). For DBP, both MAMC and MUAC were positively associated with the changes of BP values in men in Model 3. No interaction by gender was found for either SBP or DBP regardless of adjusted models. As shown in Table 4, the association of MAMC with the incidence of hypertension was statistical significant in both men and women in Model 3 (HR = 1.10, 95%CI: 1.01, 1.19 for men; HR = 1.15, 95%CI: 1.06, 1.26 for women). Stronger association with hypertension risk was found for MUAC in both men and women compared with TST. No interaction by gender was found for MAMC, MUAC or TST. In RCS analysis, although no P‐value for nonlinearity was statistically significant, J‐shaped relationships between hypertension risk and MAMC, as well as MUAC were found (Figure 2). As shown in figures, the hypertension risk did not dramatically increase until the values of MAMC and MUAC became larger than their medians. On the contrary, statistically significant increase of hypertension risk was only found in the situation of TST less than the median in men.

**TABLE 4:** Adjusted associations of hypertension incidence with per SD increase of MAMC, MUAC, and TST (no. = 3442)

	Men (n=1,504)			Women (n=1,938)			
	HR	95% CI	P‐value	HR	95% CI	P‐value	P‐value for interaction
Model 1							
MAMC	1.11	1.02, 1.21	0.017	1.17	1.07, 1.28	<0.001	0.681
MUAC	1.21	1.11, 1.32	<0.001	1.21	1.11, 1.31	<0.001	0.414
TST	1.11	1.03, 1.20	0.006	1.05	1.01, 1.19	0.023	0.402
Model 2							
MAMC	1.10	1.01, 1.20	0.031	1.18	1.08, 1.29	<0.001	0.663
MUAC	1.20	1.10, 1.31	<0.001	1.22	1.12, 1.32	<0.001	0.387
TST	1.11	1.03, 1.19	0.008	1.10	1.01, 1.19	0.021	0.403
Model 3							
MAMC	1.10	1.01, 1.19	0.037	1.15	1.06, 1.26	0.002	0.600
MUAC	1.18	1.08, 1.29	<0.001	1.19	1.09, 1.29	<0.001	0.548
TST	1.09	1.01, 1.17	0.033	1.08	0.99, 1.17	0.078	0.472

MAMC, MUAC, and TST were analyzed in separate regression models. Model 1: adjusted for age; Model 2: adjusted for smoking and alcohol drinking in addition to Model 1; Model 3: adjusted for TG, TC, HDL‐C, and FG in addition to Model 1. Abbreviations: HR, hazard ratio; 95% CI, 95% confidence interval; MAMC, mid‐upper arm muscle circumference; MUAC, mid‐upper arm circumference; TST, triceps skinfold thickness.

On the other hand, no statistically significant association between changes of MAMC with either change of BP value during the follow‐up (Table S2), or hypertension risk (Table S3) was found in the cohort analysis. Associations of changes of MUAC and TST during the follow‐up with hypertension risk were only found in men in Model 3 (HR = 1.02, 95%CI: 1.00, 1.05 for MUAC; HR = 1.01, 95%CI: 1.00, 1.02 for TST).

## 4 DISCUSSION

In summary, we found that MAMC was positively associated with both BP values and hypertension prevalence in both men and women after adjusted for biochemical confounders at baseline. In the cohort analysis, independent associations of MAMC with the change of DBP value during the follow‐up, as well as the hypertension risk were also found. Compared with TST, a stronger association of MUAC with BP values and hypertension prevalence/incidence was consistently found, which further supported the positive association of arm LBM with hypertension.

Low percentage of LBM or sarcopenia has been generally considered as a risk factor of geriatric morbidities and mortalities.^30^, ^31^, ^32^ However, recent researches obtained opposite findings that LBM was positively associated with several cardiovascular abnormalities including hypertension in populations with a broader range of age.^4^, ^5^, ^33^, ^34^ A recent cross‐sectional study from 534 Finland individuals (mean age 61 ± 3 years) demonstrated that LBM measured by bio‐impedance was a significant determinant of BP levels irrespective of age, sex, smoking and leisure‐time physical activity.^33^ Another cross‐sectional study from 3,130 Chinese people aged from 18 to 80 years old showed a positive association of LBM measured using DXA with hypertension.^9^ Consistent results were also reported in other populations of young adults, or postmenopausal women.^19^, ^35^

Up to now, all those published studies disclosing the positive relationship between LBM and BP/hypertension were cross‐sectional nature, which prevented us to define any causal relationships between them. As far as we know, our study is the first one uncovering the causality between LBM estimated by MAMC and hypertension risk using a large‐scale longitudinal database from general Chinese population. By combining both baseline and cohort analyses, our study revealed that elevated arm LBM was an independent risk factor of hypertension in Chinese populations.

Currently, several image methods such as overall body CT, and DXA emerged.^36^, ^37^ Although the body compositions can be directly and accurately estimated using these methods, disadvantages including expensive costs, complicated operations, and harmful effects on human bodies restrict them to be generally used in populations. In contrast, MAMC make it easier for different populations to estimate the LBM or muscle mass, and thus is suitable for the large‐scale population‐based studies Our study was the first attempt utilizing MAMC to estimate LBM in the Chinese population with a broad range of age. According to our findings, we thought that by utilizing such an easily obtained measurement, people could monitor their LBM more frequently, and the awareness of preventing hypertension and other CVD risks could be enhanced.

Another unique design in our study is that by using standardized regression coefficients we found that in both men and women, MUAC was stronger associated with BP and hypertension than TST. Such finding perhaps could be partially explained by the special capacity of MUAC in measuring both fat and skeletal muscle mass, and indirectly elucidated the positive effect of LBM on BP. In addition, MAMC was also stronger associated with elevated BP and hypertension than TST. Ye and associates performed a comparative analysis using z‐scored LBM indices and found that arm LBM was stronger positively associated with hypertension than fat body mass, which was in line with what we found.^9^ In accordance with findings of published studies and ours, despite that obesity is closely correlated with hypertension risk, the unfavorable effect of increased LBM cannot be neglected.

To our knowledge, the underlying mechanism responsible for the positive relationship between LBM and elevated BP or hypertension has not been fully understood. One possible underlying mechanism might be that lager LBM could result in the left ventricular hypertrophy, which thereafter increased the cardiac output and blood volume.^34^, ^38^ Simultaneously, skeletal muscle hypertrophy‐related exercise could also elevate BP value via increasing arterial stiffness and activating the sympathetic nervous system.^39^, ^40^, ^41^ On the other hand, skeletal muscle cells were also found be able to produce and secrete inflammation‐related cytokines, and consequently contributed to inflammation‐related disorders.^42^

In addition, results from RCS analysis showed a trend of J‐shaped relationship between hypertension risk and MAMC in both men and women, which as we know, has never been reported in published studies. Such J‐shaped trend suggested that there perhaps existed a threshold of LBM for triggering the risk of hypertension (eg, 23.1  and 21.0 cm of MAMC, respectively, in men and women, as shown in Figure 2), meaning that the risk of hypertension increased only in people with LBM higher than the threshold. In a previous report performed among athletes, higher BP values were observed in strength‐trained athletes than those in endurance‐trained athletes, which was probably attributed to larger LBM.^43^ This finding could support our results suggesting that cautions should be given when exploring the positive relationship between LBM and hypertension risk.

From the perspective of clinical applications of LBM, our current findings implied that MAMC, as an easier obtained and non‐invasive measurement of LBM, could be generally applied in clinical settings to predict the risk of hypertension. In addition, the capacity of LBM in predicting hypertension perhaps varied in different people, which should be well distinguished in practicing. For people with relevant lower LBM (e.g. elder people), the promotion on LBM or skeletal muscle strength seemed to be encouraged to prevent sarcopenia or frailty as previous studies showed.^44^ However, precautions on the occurrence of hypertension should be paid on people whose percentages of LBM were always extremely high, such as those frequently working on skeletal muscle hypertrophy‐related physical activities. Therefore, in the clinical practice, measurements of LBM and BP values were recommended to people with large LBM regardless of the existence of hypertension. We supposed that LBM measurement would help physicians to find out susceptible people of hypertension.

Even though associations of changes of MAMC, MUAC, and TST with hypertension risk were estimated in our study, statistical significance was only shown for MUAC and TST in men, and no interaction by gender was found. One possible reason perhaps was that in our specific population, less changes probably occurred on MAMC compared to MUAC or TST, and then the latter ones contributed more in rising the hypertension risk. On the other hand, given the limited duration of follow‐up, the range of body shape change was generally narrow, which thereafter resulted in overall smaller values of HR than those analyzed directly using measurements at baseline shown in Table 4.

Several limitations should be considered when interpreting our results. First, compared to the part of baseline analysis, the smaller sample size in cohort analysis perhaps resulted in the attenuated association of LBM measurements with both elevated BP and hypertension. Second, in our study, only MAMC was assessed as an index of LBM (upper body mainly), results perhaps would be different when using other LBM indices. For example, among more than 50 000 Scottish people, the percentage of skeletal muscle calculated using anthropometric measurements was inversely associated with BP.^10^ Genetic heterogeneity might be another reason contributing to this inconsistency. Therefore, cautions should also be paid when explaining our results in other populations with different backgrounds. Last but not least, the association of hypertension with the change of MAMC during the follow‐up was not well verified, which as we thought might be mainly resulted from the narrow range of change on body shape, especially LBM among general population. Relevant intervention studies about MAMC or other LBM measurements seem necessary. One of strengths in our study was that participants were randomly selected from 15 provinces with a broad age range, which could well represent characteristics of the general Chinese population. Secondly, we compared strengths of associations of BP values and hypertension with MAMC, MUAC and TST using standardized coefficients, and results could provide some references in the utility of these measurements.

## 5 CONCLUSIONS

MAMC was positively associated with BP values and hypertension risk independent of biochemical confounders in men and women. In addition, MUAC showed stronger association with hypertension than TST, indirectly elucidating the positive effect of LBM on BP. Findings in our study implied that we cannot neglect the capacity of LBM in predicting hypertension risk, and LBM estimates should be recommended in general health surveys or examinations.

## CONFLICTS OF INTEREST

The authors have declared that no competing interests exist.

## AUTHOR CONTRIBUTIONS

Study conception and design: Y.L and Yo.L; Analysis and interpretation of data: Y.L.; Drafting of the manuscript: Y.L; Critical revision: Y.L, YoL, and G.S. All authors read and approved the final manuscript

## Supporting information



SUPPORTING INFORMATION

## References

[1] Atkins JL , Whincup PH , Morris RW , et al. Sarcopenic obesity and risk of cardiovascular disease and mortality: a population‐based cohort study of older men. J Am Geriatr Soc. 2014;62(2):253‐260. 24428349 10.1111/jgs.12652 PMC4234002

[2] Wang M , Tan Y , Shi Y , et al. Diabetes and sarcopenic obesity: pathogenesis, diagnosis, and treatments. Front Endocrinol (Lausanne). 2020;11. 10.3389/fendo.2020.00568 PMC7477770 32982969

[3] Atkins JL , Wannamathee SG . Sarcopenic obesity in ageing: cardiovascular outcomes and mortality. Br J Nutr. 2020;124(10):1102‐1113. 32616084 10.1017/S0007114520002172

[4] Arnold M , Linden A , Clarke R , et al. Carotid intima‐media thickness but not carotid artery plaque in healthy individuals is linked to lean body mass. J Am Heart Assoc. 2019;8(15):e011919. 31364443 10.1161/JAHA.118.011919 PMC6761650

[5] Kavey RE . Left ventricular hypertrophy in hypertensive children and adolescents: predictors and prevalence. Curr Hypertens Rep. 2013;15(5):453‐457. 23893038 10.1007/s11906-013-0370-3

[6] Shea JR , Henshaw MH , Carter J , Chowdhury SM . Lean body mass is the strongest anthropometric predictor of left ventricular mass in the obese paediatric population. Cardiol Young. 2020;30(4):476‐481. 32172704 10.1017/S1047951120000311 PMC7977683

[7] Santhanam P , Sarkar S , Ahima RS . Relationship between lean body mass indices, physical activity, and systolic BP: analysis of 1999–2006 NHANES data. J Clin Hypertens (Greenwich). 2019;21(5):692‐693. 30892817 10.1111/jch.13516 PMC8030509

[8] Julius S , Majahalme S , Nesbitt S , et al. A “gender blind” relationship of lean body mass and blood pressure in the Tecumseh study. Am J Hypertens. 2002;15(3):258‐263. 11939617 10.1016/s0895-7061(01)02282-8

[9] Ye S , Zhu C , Wei C , et al. Associations of body composition with blood pressure and hypertension. Obesity (Silver Spring). 2018;26(10):1644‐1650. 30260578 10.1002/oby.22291

[10] Han TS , Al‐Gindan YY , Govan L , Hankey CR , Lean MEJ . Associations of body fat and skeletal muscle with hypertension. J Clin Hypertens (Greenwich). 2019;21(2):230‐238. 30525280 10.1111/jch.13456 PMC8030364

[11] Sizoo D , de Heide LJM , Emous M , et al. Measuring muscle mass and strength in obesity: a review of various methods. Obes Surg. 2021;31(1):384‐393. 33159294 10.1007/s11695-020-05082-2 PMC7808984

[12] Landi F , Russo A , Liperoti R , et al. Midarm muscle circumference, physical performance and mortality: results from the aging and longevity study in the Sirente geographic area (ilSIRENTE study). Clin Nutr. 2010;29(4):441‐447. 20116909 10.1016/j.clnu.2009.12.006

[13] Tian S , Xu Y . Association of sarcopenic obesity with the risk of all‐cause mortality: a meta‐analysis of prospective cohort studies. Geriatr Gerontol Int. 2016;16(2):155‐166. 26271226 10.1111/ggi.12579

[14] He L , Yang N , Wang J , et al. Mid‐arm muscle and subcutaneous fat associated with all‐cause mortality independent of BMI: a prospective cohort study. Obesity (Silver Spring). 2021;29(7):1203‐1214. 34021531 10.1002/oby.23179

[15] Noori N , Kopple JD , Kovesdy CP , et al. Mid‐arm muscle circumference and quality of life and survival in maintenance hemodialysis patients. Clin J Am Soc Nephrol. 2010;5(12):2258‐2268. 20947789 10.2215/CJN.02080310 PMC2994088

[16] Lambell KJ , Earthman CP , Tierney AC , et al. How does muscularity assessed by bedside methods compare to computed tomography muscle area at intensive care unit admission? A pilot prospective cross‐sectional study. J Hum Nutr Diet. 2021;34(2):345‐355. 32869430 10.1111/jhn.12804

[17] Carnevale V , Castriotta V , Piscitelli PA , et al. Assessment of skeletal muscle mass in older people: comparison between 2 anthropometry‐based methods and dual‐energy X‐ray absorptiometry. J Am Med Dir Assoc. 2018;19(9):793‐796. 29983360 10.1016/j.jamda.2018.05.016

[18] Miyazaki S , Hayashino S , Matsumoto I , et al. Mid‐arm muscle circumference as an indicator of exercise tolerance in chronic heart failure. Geriatr Gerontol Int. 2021;21(5):411‐415. 33821564 10.1111/ggi.14154

[19] Vaziri Y , Bulduk S , Shadman Z , et al. Lean body mass as a predictive value of hypertension in young adults. Iran J Public Health. 2015;44(12):1643‐1654. 26811815 PMC4724737

[20] Liu Y , Li Y , He J , et al. Gender stratified analyses of the association of skinfold thickness with hypertension: a cross‐sectional study in general Northeastern Chinese residents. Int J Environ Res Public Health. 2018;15(12). 10.3390/ijerph15122748 PMC6313501 30563102

[21] Brady TM . Obesity‐related hypertension in children. Front Pediatr. 2017;5:197. 28993801 10.3389/fped.2017.00197 PMC5622310

[22] Zhang B , Zhai FY , Du SF , Popkin BM . The China Health and Nutrition Survey. Obes Rev. 2014;15 suppl 1:2‐7. 10.1111/obr.12119 PMC3869031 24341753

[23] Popkin BM , Du S , Zhai F , Zhang B . Cohort Profile: the China Health and Nutrition Survey–monitoring and understanding socio‐economic and health change in China, 1989‐0011. Int J Epidemiol. 2010;39(6):1435‐1440. 19887509 10.1093/ije/dyp322 PMC2992625

[24] He L , Yang N , Wang J , et al. Mid‐Arm Muscle and subcutaneous fat associated with all‐cause mortality independent of BMI: a prospective cohort study. Obesity. 2021;29(7):1203‐1214. 34021531 10.1002/oby.23179

[25] Huang CX , Tighiouart H , Beddhu S , et al. Both low muscle mass and low fat are associated with higher all‐cause mortality in hemodialysis patients. Kidney Int. 2010;77(7):624‐629. 20072111 10.1038/ki.2009.524 PMC3155769

[26] Xue Y , Shen Q , Li C , Dai Z , He T . The Visceral Adipose Index in Relation to Incidence of Hypertension in Chinese Adults: china Health and Nutrition Survey (CHNS). Nutrients. 2020;12(3). 10.3390/nu12030805 PMC7146372 32197411

[27] Pickering TG , Hall JE , Appel LJ , et al. Recommendations for blood pressure measurement in humans and experimental animals: part 1: blood pressure measurement in humans: a statement for professionals from the subcommittee of professional and public education of the American Heart Association Council on High Blood Pressure Research. Circulation. 2005; 111(5):697‐716. 15699287 10.1161/01.CIR.0000154900.76284.F6

[28] Han T , Zhang S , Duan W , et al. Eighteen‐year alcohol consumption trajectories and their association with risk of type 2 diabetes and its related factors: the China Health and Nutrition Survey. Diabetologia. 2019;62(6):970‐980. 30923839 10.1007/s00125-019-4851-z

[29] Ren H , Zhang L , Liu Z , Zhou X , Yuan G . Sleep duration and apolipoprotein B in metabolically healthy and unhealthy overweight/obese phenotypes: a cross‐sectional study in Chinese adults. BMJ Open. 2019;9(2):e023817. 10.1136/bmjopen-2018-023817 PMC6377547 30755447

[30] Bellanti F , Romano AD , et al. Oxidative stress is increased in sarcopenia and associated with cardiovascular disease risk in sarcopenic obesity. Maturitas. 2018;109:12. 10.1016/j.maturitas.2017.12.002 29452783

[31] Rong YD , Bian AL , Hu HY , Ma Y , Zhou XZ . Study on relationship between elderly sarcopenia and inflammatory cytokine IL‐6, anti‐inflammatory cytokine IL‐10. BMC Geriatr. 2018;18(1):308. 30541467 10.1186/s12877-018-1007-9 PMC6292155

[32] Zhang X , Wang C , Dou Q , et al. Sarcopenia as a predictor of all‐cause mortality among older nursing home residents: a systematic review and meta‐analysis. BMJ Open. 2018;8(11):e021252. 10.1136/bmjopen-2017-021252 PMC6252774 30420343

[33] Korhonen PE , Mikkola T , Kautiainen H , Eriksson JG . Both lean and fat body mass associate with blood pressure. Eur J Intern Med. 2021. 10.1016/j.ejim.2021.04.025 33994250

[34] Moreno M , Puig J , Moreno‐Navarrete JM , et al. Lean mass, and not fat mass, is an independent determinant of carotid intima media thickness in obese subjects. Atherosclerosis. 2015;243(2):493‐498. 26520905 10.1016/j.atherosclerosis.2015.09.012

[35] Peppa M , Koliaki C , Boutati E , et al. Association of lean body mass with cardiometabolic risk factors in healthy postmenopausal women. Obesity (Silver Spring). 2014;22(3):828‐835. 23512933 10.1002/oby.20389

[36] Lee JJ , Pedley A , Hoffmann U , et al. Visceral and Intrahepatic Fat Are Associated with Cardiometabolic Risk Factors Above Other Ectopic Fat Depots: the Framingham Heart Study. Am J Med. 2018;131(6):e612. 10.1016/j.amjmed.2018.02.002 PMC5964004 29518370

[37] Jo A , Mainous AG . Informational value of percent body fat with body mass index for the risk of abnormal blood glucose: a nationally representative cross‐sectional study. BMJ Open. 2018;8(4):e019200. 10.1136/bmjopen-2017-019200 PMC5905746 29654009

[38] Leischik R , Spelsberg N , Niggemann H , Dworrak B , Tiroch K . Exercise‐induced arterial hypertension ‐ an independent factor for hypertrophy and a ticking clock for cardiac fatigue or atrial fibrillation in athletes?. 2014;3:105. 10.12688/f1000research.4001.1 PMC4118759 25132960

[39] Smith MM , Buffington CA , Hamlin RL , Devor ST . Relationship between muscle sympathetic nerve activity and aortic wave reflection characteristics in aerobic‐ and resistance‐trained subjects. Eur J Appl Physiol. 2015;115(12):2609‐2619. 26245524 10.1007/s00421-015-3230-9

[40] Saito M , Iwase S , Hachiya T . Resistance exercise training enhances sympathetic nerve activity during fatigue‐inducing isometric handgrip trials. Eur J Appl Physiol. 2009;105(2):225‐234. 18941773 10.1007/s00421-008-0893-5

[41] Fossum E , Hoieggen A , Reims HM , et al. High screening blood pressure is related to sympathetic nervous system activity and insulin resistance in healthy young men. Blood Press. 2004;13(2):89‐94. 15182111 10.1080/08037050310031008

[42] Sun L , Hu FB , Yu Z , et al. Lean body mass, interleukin 18, and metabolic syndrome in apparently healthy Chinese. PLoS One. 2011;6(3):e18104. 21437204 10.1371/journal.pone.0018104 PMC3060923

[43] Berge HM , Isern CB , Berge E . Blood pressure and hypertension in athletes: a systematic review. Br J Sports Med. 2015;49(11):716‐723. 25631543 10.1136/bjsports-2014-093976

[44] Bai T , Fang F , Li F , et al. Sarcopenia is associated with hypertension in older adults: a systematic review and meta‐analysis. BMC Geriatr. 2020;20(1):279. 32762638 10.1186/s12877-020-01672-y PMC7409686

