# Supplementary material for: A prospective cohort study on the association of lean body mass estimated by mid‐upper arm muscle circumference with hypertension risk in Chinese residents
Source: J Clin Hypertens (Greenwich). 2022 Feb 16;24(3):329–38. doi: 10.1111/jch.14412 (PMC8925010; doi:10.1111/jch.14412)
Supplement: Supplementary file 1 — SUPPORTING INFORMATION [file JCH-24-329-s001.docx]

**Supplemental Tables**

| **Table S1.** Adjusted associations of changes of blood pressure with per SD increase of MAMC, MUAC and TST(n=3,261) | | | | | | | |
| --- | --- | --- | --- | --- | --- | --- | --- |
|  | Men (n=1,424) | | | Women (n=1,837) | | | P-value for interaction |
|  | BP (mmHg) | 95% CI | P-value | BP (mmHg) | 95% CI | P-value |  |
| Change of SBP | | | | | | | |
| Model 1 | | | | | | | |
| MAMC | 0.227 | -0.595, 1.049 | 0.588 | 0.061 | -0.759, 0.879 | 0.885 | 0.789 |
| MUAC | 0.956 | 0.105, 1.806 | 0.028 | 0.271 | -0.501, 1.043 | 0.491 | 0.270 |
| TST | 0.898 | 0.127, 1.669 | 0.023 | 0.330 | -0.439, 1.100 | 0.400 | 0.333 |
| Model 2 | | | | | | | |
| MAMC | 0.150 | -0.676, 0.977 | 0.722 | 0.101 | -0.718, 0.920 | 0.809 | 0.857 |
| MUAC | 0.867 | 0.005, 1.729 | 0.049 | 0.293 | -0.479, 1.065 | 0.457 | 0.316 |
| TST | 0.864 | 0.091, 1.637 | 0.029 | 0.312 | -0.458, 1.081 | 0.427 | 0.351 |
| Model 3 | | | | | | | |
| MAMC | 0.166 | -0.664, 0.996 | 0.695 | 0.050 | -0.775, 0.875 | 0.906 | 0.777 |
| MUAC | 0.833 | -0.048, 1.713 | 0.064 | 0.283 | -0.508, 1.074 | 0.483 | 0.276 |
| TST | 0.788 | 0.001, 1.575 | 0.050 | 0.354 | -0.429, 1.136 | 0.375 | 0.355 |
| Change of DBP | | | | | | | |
| Model 1 | | | | | | | |
| MAMC | 0.601 | 0.020, 1.182 | 0.043 | 0.525 | -0.010, 1.060 | 0.054 | 0.801 |
| MUAC | 0.834 | 0.232, 1.435 | 0.007 | 0.235 | -0.271, 0.740 | 0.363 | 0.085 |
| TST | 0.269 | -0.277, 0.816 | 0.334 | -0.319 | -0.822, 0.184 | 0.214 | 0.074 |
| Model 2 | | | | | | | |
| MAMC | 0.556 | -0.028, 1.140 | 0.062 | 0.541 | 0.006, 1.077 | 0.048 | 0.863 |
| MUAC | 0.773 | 0.163, 1.382 | 0.013 | 0.243 | -0.263, 0.748 | 0.347 | 0.105 |
| TST | 0.234 | -0.314, 0.782 | 0.402 | -0.326 | -0.830, 0.178 | 0.204 | 0.083 |
| Model 3 | | | | | | | |
| MAMC | 0.605 | 0.019, 1.191 | 0.043 | 0.563 | 0.025, 1.102 | 0.040 | 0.771 |
| MUAC | 0.817 | 0.195, 1.439 | 0.010 | 0.319 | -0.198, 0.835 | 0.226 | 0.080 |
| TST | 0.199 | -0.358, 0.756 | 0.484 | -0.260 | -0.771, 0.251 | 0.318 | 0.088 |
| MAMC, MUAC and TST were analyzed in separate regression models. Model 1: adjusted for age; Model 2: adjusted for smoking and alcohol drinking in addition to Model 1; Model 3: adjusted for TG, TC, HDL-C, and FG in addition to Model 1. Abbreviations: BP: blood pressure; 95% CI: 95% confidence interval; SBP: systolic blood pressure; DBP: diastolic blood pressure; MAMC: mid-upper arm muscle circumference; MUAC: mid-upper arm circumference; TST: triceps skinfold thickness. | | | | | | | |

| **Table S2.** Adjusted associations of changes of blood pressure with changes of MAMC, MUAC and TST (n=3,261) | | | | | | | |
| --- | --- | --- | --- | --- | --- | --- | --- |
|  | Men (n=1,424) | | | Women (n=1,837) | | | P-value for interaction |
|  | BP (mmHg) | 95% CI | P-value | BP (mmHg) | 95% CI | P-value |  |
| Change of SBP | | | | | | | |
| Model 1 | | | | | | | |
| Change of MAMC | 0.067 | -0.107, 0.240 | 0.450 | 0.086 | -0.086, 0.259 | 0.328 | 0.861 |
| Change of MUAC | 0.083 | -0.134, 0.300 | 0.454 | 0.069 | -0.133, 0.271 | 0.501 | 0.958 |
| Change of TST | -0.010 | -0.092, 0.072 | 0.811 | -0.026 | -0.109, 0.057 | 0.537 | 0.798 |
| Model 2 | | | | | | | |
| Change of MAMC | 0.066 | -0.108, 0.239 | 0.457 | 0.076 | -0.097, 0.249 | 0.389 | 0.858 |
| Change of MUAC | 0.086 | -0.132, 0.303 | 0.440 | 0.062 | -0.139, 0.264 | 0.544 | 0.962 |
| Change of TST | -0.008 | -0.090, 0.074 | 0.848 | -0.022 | -0.105, 0.061 | 0.600 | 0.800 |
| Model 3 | | | | | | | |
| Change of MAMC | 0.071 | -0.103, 0.245 | 0.426 | 0.089 | -0.083, 0.262 | 0.310 | 0.859 |
| Change of MUAC | 0.093 | -0.125, 0.311 | 0.402 | 0.069 | -0.133, 0.271 | 0.504 | 0.943 |
| Change of TST | -0.008 | -0.091, 0.074 | 0.848 | -0.029 | -0.112, 0.054 | 0.495 | 0.776 |
| Change of DBP | | | | | | | |
| Model 1 | | | | | | | |
| Change of MAMC | -0.001 | -0.124, 0.122 | 0.989 | 0.023 | -0.090, 0.136 | 0.689 | 0.866 |
| Change of MUAC | 0.117 | -0.037, 0.270 | 0.136 | 0.071 | -0.061, 0.203 | 0.290 | 0.525 |
| Change of TST | 0.054 | -0.004, 0.112 | 0.068 | 0.021 | -0.033, 0.076 | 0.439 | 0.379 |
| Model 2 | | | | | | | |
| Change of MAMC | 0.002 | -0.121, 0.125 | 0.976 | 0.020 | -0.093, 0.133 | 0.733 | 0.871 |
| Change of MUAC | 0.122 | -0.031, 0.276 | 0.118 | 0.069 | -0.063, 0.201 | 0.308 | 0.514 |
| Change of TST | 0.055 | -0.004, 0.112 | 0.066 | 0.022 | -0.032, 0.077 | 0.418 | 0.376 |
| Model 3 | | | | | | | |
| Change of MAMC | -0.005 | -0.128, 0118 | 0.941 | 0.029 | -0.084, 0.142 | 0.618 | 0.785 |
| Change of MUAC | 0.117 | -0.037, 0.271 | 0.138 | 0.069 | -0.063, 0.202 | 0.303 | 0.556 |
| Change of TST | 0.056 | -0.002, 0.115 | 0.058 | 0.016 | -0.004, 0.071 | 0.559 | 0.326 |
| MAMC, MUAC and TST were analyzed in separate regression models. Model 1: adjusted for age; Model 2: adjusted for smoking and alcohol drinking in addition to Model 1; Model 3: adjusted for TG, TC, HDL-C, and FG in addition to Model 1. Abbreviations: BP: blood pressure; 95% CI: 95% confidence interval; SBP: systolic blood pressure; DBP: diastolic blood pressure; MAMC: mid-upper arm muscle circumference; MUAC: mid-upper arm circumference; TST: triceps skinfold thickness. | | | | | | | |

| **Table S3.** Adjusted associations of hypertension incidence with changes of MAMC, MUAC and TST (n=3,442) | | | | | | | |
| --- | --- | --- | --- | --- | --- | --- | --- |
|  | Men (n=1,504) | | | Women (n=1,938) | | | P-value for interaction |
|  | HR | 95% CI | P-value | HR | 95% CI | P-value |  |
| Model 1 | | | | | | | |
| Change of MAMC | 1.00 | 0.99, 1.02 | 0.720 | 1.00 | 0.98, 1.02 | 0.750 | 0.854 |
| Change of MUAC | 1.02 | 1.00, 1.05 | 0.039 | 1.02 | 0.99, 1.04 | 0.171 | 0.430 |
| Change of TST | 1.01 | 1.00, 1.02 | 0.044 | 1.01 | 1.00, 1.02 | 0.191 | 0.554 |
| Model 2 | | | | | | | |
| Change of MAMC | 1.00 | 0.99, 1.02 | 0.709 | 1.00 | 0.98, 1.02 | 0.899 | 0.883 |
|  |  |  |  |  |  |  |  |
| Change of MUAC | 1.02 | 1.00, 1.05 | 0.032 | 1.01 | 0.99, 1.04 | 0.234 | 0.449 |
| Change of TST | 1.01 | 1.00, 1.02 | 0.039 | 1.01 | 1.00, 1.02 | 0.174 | 0.552 |
| Model 3 | | | | | | | |
| Change of MAMC | 1.00 | 0.99, 1.02 | 0.770 | 1.00 | 0.98, 1.02 | 0.781 | 0.875 |
| Change of MUAC | 1.02 | 1.00, 1.05 | 0.036 | 1.02 | 1.00, 1.04 | 0.136 | 0.485 |
| Change of TST | 1.01 | 1.00, 1.02 | 0.032 | 1.01 | 1.00, 1.02 | 0.124 | 0.614 |
| MAMC, MUAC and TST were analyzed in separate regression models. Model 1: adjusted for age; Model 2: adjusted for smoking and alcohol drinking in addition to Model 1; Model 3: adjusted for TG, TC, HDL-C, and FG in addition to Model 1. Abbreviations: HR: hazard ratio; 95% CI: 95% confidence interval; MAMC: mid-upper arm muscle circumference; MUAC: mid-upper arm circumference; TST: triceps skinfold thickness. | | | | | | | |
